# Stockpiling Ventilators for Influenza Pandemics

**DOI:** 10.3201/eid2306.170434

**Published:** 2017-06

**Authors:** Martin I. Meltzer, Anita Patel

**Affiliations:** Centers for Disease Control and Prevention, Atlanta, Georgia, USA

**Keywords:** Ventilators, optimization, pandemic, influenza, viruses, Texas, United States, respiratory infections, stockpiles, surge capacity

For decades, public health officials have been well aware of the importance of planning and preparing for the next influenza pandemic (*1*). An open-access, spreadsheet-based tool, FluSurge 2.0, enables public health officials and hospital administrators to estimate the potential surge in demand that inevitably will occur during the next influenza pandemic. The tool provides estimates for hospital-based services, such as intensive care unit beds and mechanical ventilators (*2*,*3*). Any influenza pandemic has the potential to overwhelm existing hospital capacities, supplies, and readily available means of resupply. Thus, all capable public health services should develop and carry out plans to stockpile critical resources, such as mechanical ventilators, needed to support patients who become severely ill from pandemic influenza (*4*). A major challenge to planning is determining the amount of ventilators to stockpile and considering how to manage them so they are effectively used during the pandemic response.

In this issue (*5*), Huang et al. provide a valuable addition to public health preparedness by presenting a planning tool to help public health officials consider the optimal size of a stockpile of mechanical ventilators, compiled on the basis of data from Texas, USA. Users of their tool also can evaluate where such a stockpile should be stored: a central location, or prepositioned strategically in hospital facilities. To determine how many ventilators to stockpile and where to stockpile them, the authors explicitly include in their tool key factors such as timing of a pandemic’s peak locally (not all regions will experience simultaneous peak demands for ventilators); wastage (ventilators not sent to where they are needed, when they are most needed, and/or cannot be used); and expected unmet demand for mechanical ventilators (i.e., when a hospital has more patients who need mechanical ventilation than available ventilators).

All mathematical models have limitations, and some important practical problems related to ventilator preparedness are beyond the reasonable scope of the model by Huang et al. For example, hospitals must accept responsibility for the costs and resources needed to manage and maintain an excess of ventilators that are likely to be unused in the absence of pandemic-related surges in demand for such resources. The authors mention that a potential benefit of stockpiling ventilators at hospitals is to facilitate staff training; however, only a few (1–3) ventilators would be needed to support training needs. Also, once stockpiles are established, the costs of replenishing inventory over time or replacing products to meet changing technology are not considered. The model also assumes that stockpiled ventilators will not be used for noninfluenza patients. In reality, stockpiled ventilators are likely to be simpler ventilators that can be used on more stable patients, thus freeing up other sophisticated ventilators for patients requiring greater respiratory support.

An important aspect of interpreting the results from the model of Huang et al. is the problem of expected unmet demand. There is an upper limit to the number of additional ventilators that any hospital can absorb and use to successfully help treat acutely ill patients needing mechanical ventilation. This limit is determined in large part by the number of trained staff—particularly respiratory therapists, nurses, and technicians—available to ventilate and monitor patients (*6*). That is, the number of machines is less of a constraint than is availability of trained personnel. Huang et al. allow for expected unmet needs, setting a default value of an acceptable level of 5 patients unable to receive mechanical ventilation at any given time. Assuming a moderate, 2009-type influenza pandemic, the authors estimate a 30% chance of this expected unmet need occurring. To meet this level of unmet demand in Texas, planning for a moderate or a severe pandemic requires stockpiling as few as 1,172 or as many as 15,697 ventilators, respectively. However, actually deploying and using such high volumes of mechanical ventilators would be challenging in terms of having enough hospital space and staff to support additional ventilator use. Thus, during moderate and severe pandemics, a higher level of unmet demand might need to be expected. Attending physicians will have to determine who gets access to the limited number of ventilators and who does not. Only a small number of studies describe how physicians might make such allocation decisions for critical, scarce resources (i.e., triage or prioritization) and how they would explain such decisions to the patients and their families (*7*–*9*).

Tools of the type produced by Huang et al. are essential to adequately plan and optimize the use of stockpiled resources during the next influenza pandemic. Such tools, however, are just one part of systematic planning, which should include elements such as the number to be stockpiled, how and where stockpiles should be held, maintenance of stockpiles, conditions for release, considerations for use, and what to do when stockpiles are insufficient to adequately meet surges in demand.
